# Time course of nocturnal cough and wheezing in children with acute bronchitis monitored by lung sound analysis

**DOI:** 10.1007/s00431-019-03426-4

**Published:** 2019-07-18

**Authors:** Ulrich Koehler, Olaf Hildebrandt, Patrick Fischer, Volker Gross, Keywan Sohrabi, Nina Timmesfeld, Saskia Peter, Christof Urban, Jens-Oliver Steiß, Stephan Koelsch, Sebastian Kerzel, Andreas Weissflog

**Affiliations:** 1 Department of Internal Medicine, Pneumology, Intensive Care and Sleep Medicine, University Hospital of Marburg and Gießen, Philipps-Universität, Baldingerstrasse 1, 35043 Marburg, Germany; 2 Faculty of Health Sciences, University of Applied Sciences, Gießen, Germany; 3 Department of Medicine, Institute of Medical Biometry and Epidemiology, Philipps-University Marburg, Marburg, Germany; 4 Department of Pediatric Pneumology and Allergy, University Children’s Hospital Regensburg, St. Hedwig Campus, Regensburg, Germany; 5 Alpenklinik Santa Maria, Oberjoch, Germany; 6 Division of Pediatric Pulmonology and Allergy, Department of Pediatrics and Neonatology, University Hospital of Marburg and Gießen, Gießen, Germany; 7 CHC Medical Affairs, Sanofi-Aventis Deutschland GmbH, Frankfurt am Main, Germany; 8 Clinical Research Department, Thora Tech GmbH, Gießen, Germany

**Keywords:** cough, wheezing, acute bronchitis, acoustic long-term recording, time course

## Abstract

Cough and wheezing are the predominant symptoms of acute bronchitis. Hitherto, the evaluation of respiratory symptoms was limited to subjective methods such as questionnaires. The main objective of this study was to objectively determine the time course of cough and wheezing in children with acute bronchitis. The impact of nocturnal cough on parent’s quality of life was assessed as secondary outcome. In 36 children (2–8 years), the frequency of nocturnal cough and wheezing was recorded during three nights by automated lung sound monitoring. Additionally, parents completed symptom logs, i.e., the Bronchitis Severity Score (BSS), as well as the Parent-proxy Children’s Acute Cough-specific Quality of Life Questionnaire (PAC-QoL). During the first night, patients had 34.4 ± 52.3 (mean ± SD) cough epochs, which were significantly reduced in night 5 (13.5 ± 26.5; *p* < 0.001) and night 9 (12.8 ± 28.1; *p* < 0.001). Twenty-two patients had concomitant wheezing, which declined within the observation period as well. All subjective parameters (BSS, Cough log and PAC-QoL) were found to be significantly correlated with the objectively assessed cough parameters.

*Conclusion*: Long-term recording of cough and wheezing offers a useful opportunity to objectively evaluate the time course of respiratory symptoms in children with acute bronchitis. To assess putative effects of pharmacotherapy on nocturnal bronchitis symptoms, future studies in more homogeneous patient groups are needed.

**Table:** 

**What is Known:** • *Cough and wheezing are the predominant symptoms of acute bronchitis*. • *There is a diagnostic gap in long-term assessment of these respiratory symptoms, which needs to be closed to optimize individual therapies*. **What is New:** • *Long-term recording of nocturnal cough and wheezing allows for objective evaluation of respiratory symptoms in children with acute bronchitis and provides a tool to validate the efficacy of symptomatic bronchitis therapies*.

**What is Known:**

• *Cough and wheezing are the predominant symptoms of acute bronchitis*.

• *There is a diagnostic gap in long-term assessment of these respiratory symptoms, which needs to be closed to optimize individual therapies*.

**What is New:**

• *Long-term recording of nocturnal cough and wheezing allows for objective evaluation of respiratory symptoms in children with acute bronchitis and provides a tool to validate the efficacy of symptomatic bronchitis therapies*.

## Introduction

Infections of the bronchial system occur frequently, particularly in children and adolescents [1, 10]. More than one-third of children with acute bronchitis are presented to a general practitioner or primary care pediatrician, bearing a great clinical and health economical relevance. Symptoms of airway infections sometimes persist for more than 10 days and cough often lasts up to 3 weeks [30]. In children, acute bronchitis usually is triggered by viral infection. Symptoms include cough, wheezing, fever, headache, and a general feeling of sickness [16]. Cough is a very frequent symptom associated with respiratory tract infection, and it might deeply affect the quality of life of both children and parents [22, 25].

Acute bronchitis is an inflammation of the lower airways, most frequently due to viral infection, leading to enhanced mucus production and coughing [16]. In many cases, acute bronchial obstruction is part of the clinical picture, causing an expiratory wheezing which can be recognized by bare ear or auscultation by physical examination. Important criteria for the classification as “mere bronchitis” are absence of (i) evidence of pneumonia and (ii) chronic, recurrent symptoms. Due to this nature, “acute bronchitis” is hence primarily a clinical diagnosis with a strong emphasis on the present history and the physical examination. Within the framework of evidence-based medicine, health authorities increasingly demand proof of effectiveness of cough-related therapy, and there is still an on-going debate in the scientific community how to objectively assess the putative clinical benefit of antitussive treatments in children [7, 27]. Assessment of respiratory symptoms like cough and wheezing so far is mostly subjective and based on qualitative description of the symptoms by the patient or a parent. Manual counting of cough and wheezing over a number of hours, however, is not feasible for a large number of patients. Consequently, there is a necessity for objective assessment technologies like automated cough and wheezing monitoring [21]. In addition to clinical use, there is a certain need for objective assessment of symptoms in the context of bronchitis therapy, e.g., to evaluate antitussive treatments. Cough monitoring systems are increasingly being used as primary end-points in clinical trials. They are in general ambulatory devices that consist of wired microphones and recording device which can be attached to the patient’s body. However, these systems so far have not been used in children to monitor nocturnal cough and wheezing objectively. Accordingly, data on their suitability for long-term monitoring of respiratory symptoms in children are missing.

In the present study, automated detection and analysis of adventitious lung sounds has been carried out with the LEOSound Lung Sound Monitor to answer two main questions:

1. Is the method suitable to describe the time course of cough and wheezing in acute bronchitis?
2. Is the method suitable for repeated long-term recording of lung sound in young children in terms of acceptance and compliance?

Additionally, impact of cough on parent’s quality of life has been estimated by using the validated PAC-QoL questionnaire.

## Methods

The presented study was designed as a multicenter study, involving the University Hospital Giessen and Marburg, University Hospital Regensburg, and the Alpenklinik Santa Maria in Oberjoch.

### Study design

We established a 10-day study protocol in which repeated nocturnal lung sound recordings were performed. Study participants were recruited from children with acute bronchitis who had been admitted to the inpatient pediatric pulmonary departments of the respective study centers. Decision on admission was completely independent of the study.

Inclusion criteria were as follows: diagnosis of acute bronchitis, age between 2 and 8 years or a body weight of more than 10 kg (according to intended use; predefined condition for application by the manufacturer of the Lung Sound-Recorder), and a Bronchitis Severity Score (BSS) of five points or more. Children with chronic respiratory diseases or with onset of symptoms more than 72 h ago were excluded from the study. There were no restrictions to medication.

After recruitment, which also included an anamnesis and an introduction to the recording devices and the questionnaires, the first lung sound recording started on the evening of day one. At that time, all patients were still in hospital. The same procedure was repeated on days 5 and 9 of the study in an inpatient or outpatient (in case of patient was discharged) setting. In case of subsequent ambulatory patients, the second and third measurements were performed at home assisted by on site study staff. On the morning after lung sound recording, parents were asked to answer the cough log and the validated Parent-proxy Children’s Acute Cough-specific Quality of Life Questionnaire (PAC-QoL) for the three respective nights. BSS was taken on site by a member of the study staff. The procedures of the study are chronologically shown in Table 1.

**Table 1:** Study protocol with questionnaires and recordings as well as responsibilities

Day	Questionnaires and recordings	Involved persons
1	-Anamnesis	Physician
	-1. Bronchitis Severity Score (BSS)	Study staff
	-1. Nocturnal lung sound recording	Parents
2	-1. Cough log	Parents
	- 1. PaC-QoL	Parents
5	**-** 2. Nocturnal lung sound recording	Parents
6	- 2. Cough log	Parents
	- 2. PaC-QoL	Parents
	- 2. BSS	Study staff
9	- 3. Nocturnal lung sound recording	Parents
10	- 3. Cough log	Parents
	- 3. PaC-QoL	Parents
	- 3. BSS	Study staff

### Lung sound recording and analysis

**Fig. 1:**
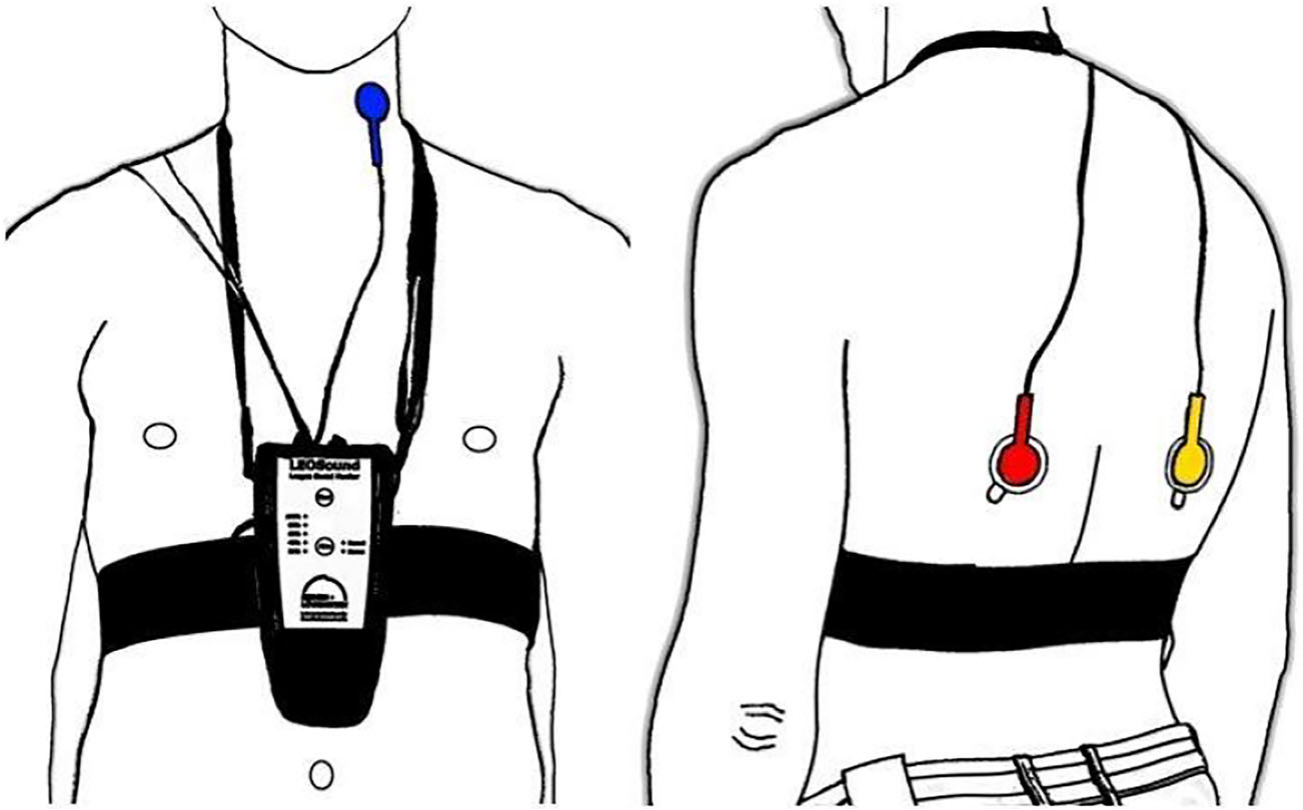
LEOSound recorder and microphone placement at the trachea (blue, left) and the chest (red and yellow, right) of the patient. The back microphones were applied with adhesive pads to the inferior left and right shoulder blade (scapula). The ideal position was identified by auscultation

LEOSound Lung-Sound-Monitor (Löwenstein Medical GmbH & Co. KG, Bad Ems, Germany) is a mobile device validated for automatic long-term recording and analysis of normal and adventitious respiratory sounds like cough and wheezing in adults and children. The system automatically detects cough and wheezing up to 24 h and can be used either in the hospital or at the patient’s home. Sound is recorded with three bio-acoustical sensors placed at the trachea and the back of the patients (Fig. 1). In addition, an ambient microphone is integrated in the LEOSound device. Thus, it is possible to differentiate lung sounds from speech and other ambient sounds. The devices were programmed in advance for every patient. The related software contains automated algorithms for cough and wheezing detection. The cough detection algorithm achieves a sensitivity and specificity of 93% and 99% respectively. Both sensitivity and specificity of wheezing detection algorithm are above 95% [12].

Nocturnal lung sound recordings lasted 10 h and started at 08:00 p.m. of each recording day. All recordings were manually validated to ensure the results did not contain any false positive results and the algorithm missed no cough event. Manual evaluation also contained a classification of all cough events in productive and non-productive cough. Studies have shown the ability of healthcare professionals to distinguish between productive and non-productive cough with a good accuracy by listening to the characteristic cough sound [28]. All cough events were then assigned to cough epochs, which were classified as productive if they contained at least one productive cough event. According to the European Respiratory Society, cough epochs are defined as at least two consecutive cough events with a maximum distance of 2 s between them [21]. To analyze the course of cough during the night, cough epochs were combined to 10-min windows.

Nocturnal wheezing was evaluated by calculating the wheezing rate and then assigning these wheezing events to wheezing phases. We defined wheezing phases as a minimum of 4 min with a wheezing rate above 2% and a gap without wheezing not longer than 1 min. In addition, results were validated by medical experts.

### Questionnaires and scores

We used the Bronchitis Severity Score (BSS) to assess the clinical severity of obstructive bronchitis in a standardized fashion [15, 20]. Physicians had to score five bronchitis symptoms: mucus production, cough, chest pain, crackles, and dyspnoea. The scores were rated on a 5-point scale from not present (0 points) to very severe (5 points). A BSS of 5 points or more was defined as moderate bronchitis. BSS was determined on day 1, day 6, and day 10 of the study.

To address the impact of cough on the patient’s quality of life we used the Parent-proxy Children’s Acute Cough-specific Quality of Life Questionnaire (PAC-QoL)*.* This validated questionnaire was completed by the parents and contains 16 questions from three different domains. The questions address physical well-being, social well-being, and psychological well-being and have to be answered on a 7-point scale [2].

To evaluate the subjectively perceived characteristics of child’s cough, parents filled out a cough log after every measuring day. The parameters cough frequency, cough characteristics, influence of coughing on sleep of the child, and influence of coughing on sleep of the guardians on a scale of 0–3 (0 = no expression/influence/frequency, 3 = strong expression/influence/frequency) were documented.

## Statistics

Statistical analysis was performed by using R version 3.4.0. For descriptive statistics, mean and standard deviation were calculated for the respective parameters.

To compare the different recording nights, we used Friedman’s test, which is the non-parametric equivalent to the analysis of variance for repeated measures. Correlation was assessed by using Pearson correlation. Results with a *p* value ≤ 0.05 were considered as significant.

## Results

### Patient recruitment

We included 44 children, comprising eight dropouts. Main reasons for dropout were refusal of the measurement device by the child (six) and a worsening of the health status (two). Hence, data of 36 patients were analyzed in detail. Chest x-ray were done in 13 patients; 19 patients were girls. Mean age of our population was 4.3 ± SD 1.84 years with a mean BMI of 15.6 ± 1.22 kg/m^2^. Anthropometric data are listed in Table 2. First symptoms occurred about 40 h (39.9 ± 19.5) before inclusion. Mean BSS taken at the inclusion was 7.5 ± 3.0 points with a moderate to severe cough attribute score of 2.4 ± 0.8 points. Only nine out of 36 patients received no treatment. All other patients received different medication, which is shown in Table 3.

**Table 2:** Anthropometric data of study population

	*N* = 36
Age (years)	4.30 ± 1.84
Gender (% male)	47.2
Height (*z* score)	− 0.019 ± 1.058
Weight (*z* score)	0.073 ± 0.97
BMI (*z* score)	− 0.066 ± 0.909

**Table 3:** Medication of study population

Medication	
No medication	9
Antibiotics	2
Salbutamol (inhalation)	21
Ipratropiumbromide (inhalation)	21
Prednisolone (intravenously)	15
Secretolytics	3
Antitussives	1
NaCl 0.9% (inhalation)	16
Supplemental oxygen	11

### Cough and wheezing

A total of 2.248 cough epochs were detected in all patients throughout the three recording nights. Most of these epochs (1.299 epochs) occurred during the first night, while nights 5 and 9 had similar and significantly less (*p* = 0.00024) number of cough epochs with 490 and 459 epochs respectively (Table 4).

**Table 4:** Cough parameters for each recording night (mean ± SD)

	Night 1	Night 5	Night 9	
	Mean ± SD	Mean ± SD	Mean ± SD	*p*
Cough epochs (productive cough)	9.44 ± 19.4	3.89 ± 10.3	0.89 ± 1.92	0.0027
Cough epochs (non-productive cough)	24.9 ± 37.4	9.58 ± 18.7	11.9 ± 27.8	0.003
Cough epochs (total)	34.4 ± 52.3	13.5 ± 26.5	12.8 ± 28.1	< 0.001

**Fig. 2:**
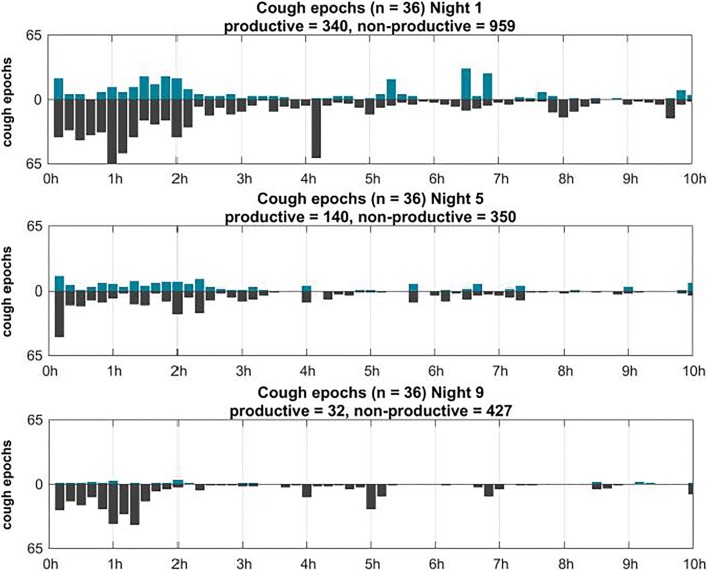
Distribution of productive (blue) and non-productive (gray) cough epochs during each recording night

**Fig. 3:**
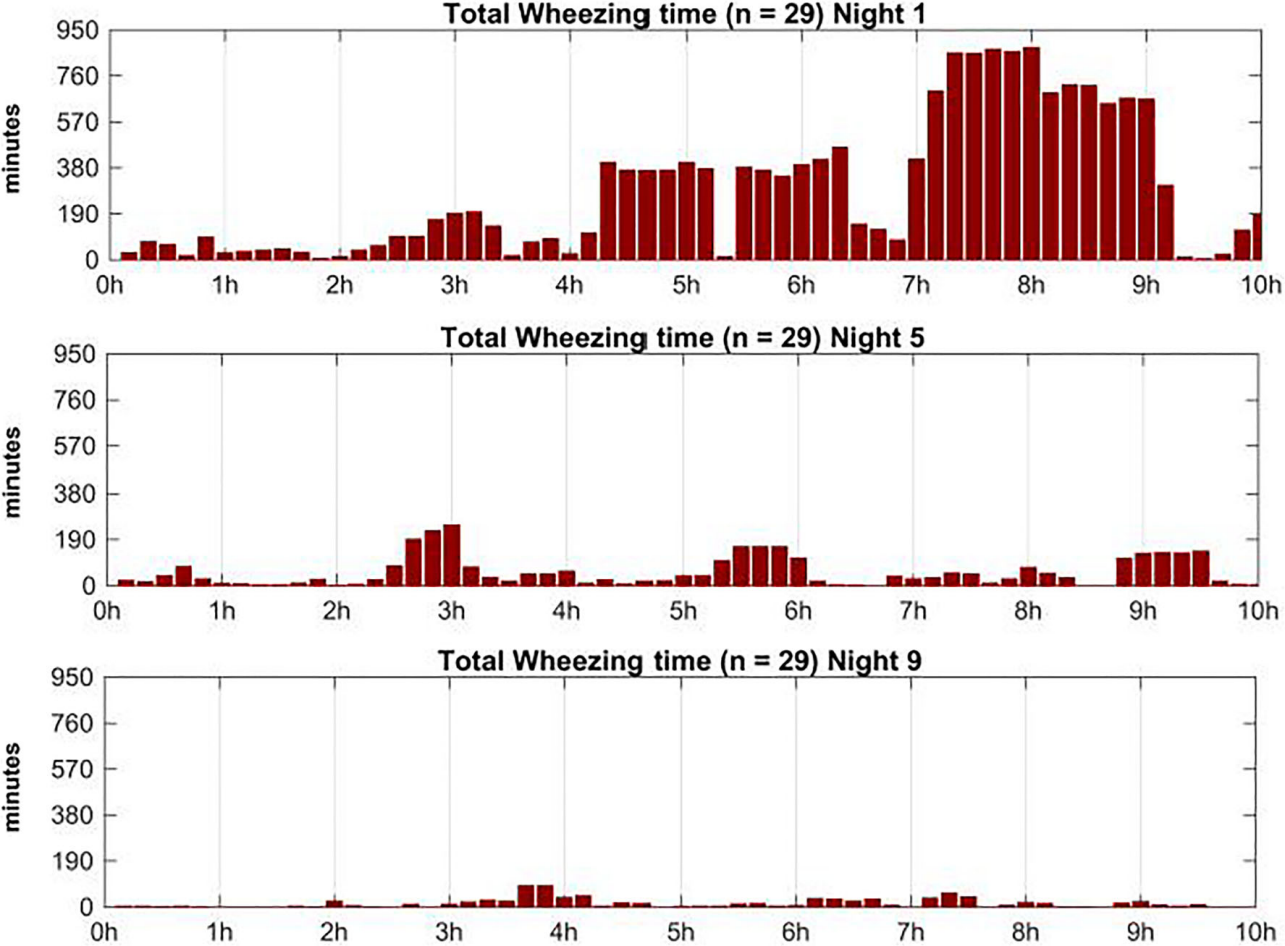
Cumulative wheezing time in minutes and its distribution over the three recording nights

In particular, productive cough decreased during the course of the study. Non-productive cough slightly increased from 350 in the second night of recording to 427 during the last night of recording (Fig. 2). Most coughing occurred during the first 3.5 h of each respective night. Wheezing was found in 29 out of 36 patients and was present during the whole night. Wheezing intensity increased during the night and had its peak in the early morning hours. Total amount of wheezing decreased throughout the study (Fig. 3).

### Questionnaires and scores

The cough log score significantly decreased during the study, while the patient’s quality of life significantly increased. Table 5 shows the descriptive values of the parent questionnaires. This table also includes a comparison between day 1 and day 6/day 10.

**Table 5:** Descriptive values (mean ± sd) of the parent filled questionnaires “Cough log” and “PaC-QoL”

						Comparison	
	Day 2	Day 6	Day 10			D2 with D6	D2 with D10
	Mean ± SD	Mean ± SD	Mean ± SD	*p*		Mean ± SD	Mean ± SD
Cough log	8.37 ± 3.42	6.47 ± 3.47	5.61 ± 3.05	< 0.001	Cough log	− 1.83 ± 4.65	− 2.71 ± 4.03
PAC-QoL	68.1 ± 19.6	80.3 ± 25.2	93.5 ± 18.8	< 0.001	PAC-QoL	12.2 ± 25.5	25.3 ± 24.5

Bronchitis Severity Score assessed by physician decreased significantly. A detailed analysis of the cough related questions of the BSS showed a significant decrease for both cough and mucus (Table 6).

**Table 6:** Descriptive values (mean ± SD) of Bronchitis Severity Score (BSS) assessed by physician

						Comparison	
	Day 1	Day 6	Day 10			D1 with D6	D1 with D10
	Mean ± SD	Mean ± SD	Mean ± SD	*p*		Mean ± SD	Mean ± SD
BSS total	7.54 ± 3.01	3.69 ± 2.13	1.56 ± 1.88	< 0.001	BSS total	− 3.86 ± 3.53	− 6.00 ± 3.70
BSS cough	2.40 ± 0.81	1.46 ± 0.61	0.82 ± 0.83	< 0.001	BSS cough	− 0.94 ± 0.87	− 1.59 ± 1.18
BSS mucus	1.80 ± 0.96	1.29 ± 0.75	0.50 ± 0.79	< 0.001	BSS mucus	− 0.51 ± 1.07	− 1.29 ± 1.19

### Comparison of subjective and objective assessments

**Fig. 4:**
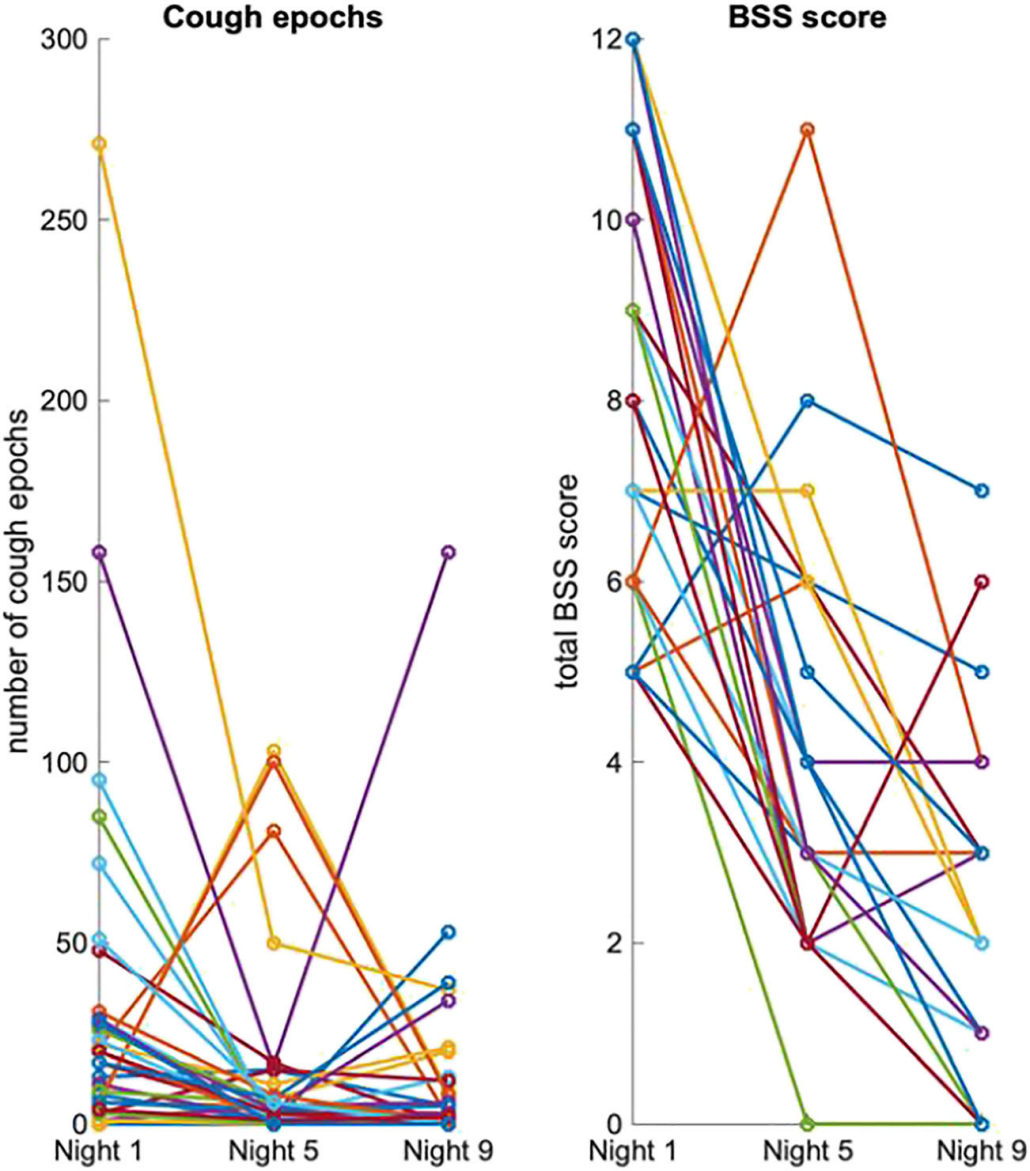
Individual course of cough epochs (left) assessed by objective lung sound analysis and total BSS score assessed by physician (right)

All three subjectively assessed parameters (BSS, Cough log, and PaC-QoL) were found to be significantly correlated with objectively assessed cough parameters. Cough log score was found to be significantly correlated with the amount of non-productive cough epochs (*r* = 0.61, *p* < 0.001), as well as with the total amount of cough epochs (*r* = 0.61, *p* < 0.001). Wheezing was significantly correlated with BSS total score (*r* = 0.29, *p* = 0.003, see Table 7). Questionnaire scores showed significant correlation with BSS assessed by physician (Tables 7 and 8). The individual course of cough epochs and BSS during the study is illustrated in Fig. 4.

**Table 7:** Correlation between subjective and objective assessments. (*r* = correlation coefficient; *p* = significance; [] = 95% confidence interval). Significant results are marked in italic

	Cough log score	BSS total score	PAC-QoL score
Cough epochs (productive cough)	[0.27; 0.58]	[0.2; 0.53]	[−0.44; −0.09]
	*r* = *0.44, p* < *0.001*	*r* = *0.38, p* < *0.001*	*r* = − *0.27*, *p* = *0.004*
Cough epochs (non-productive cough)	[0.47; 0.72]	[0.17; 0.51]	[− 0.58; − 0.28]
	*r* = *0.61*, *p* < *0.001*	*r* = *0.35*, *p* < *0.001*	*r* = − *0.44*, *p* < *0.001*
Cough epochs (total cough)	[0.48; 0.72]	[0.23; 0.55]	[− 0.57; − 0.26]
	*r* = 0*.61*, *p* < *0.001*	*r* = *0.4*, *p* < *0.001*	*r* = − *0.43*, *p* < *0.001*
Wheezing rate	[− 0.01; 0.36]	[0.1; 0.46]	[− 0.37; 0]
	*r* = 0.18, *p* = 0.068	*r* = *0.29, p* = *0.003*	*r* = − 0.19, *p* = 0.055

**Table 8:** Correlation between cough questionnaires and BSS assessed by physician (*r* = correlation coefficient; *p* = significance; [] = 95% confidence interval). Significant results are marked in italic

	BSS total score	PAC-QoL score
Cough log score	[0.32; 0.62]	[− 0.66; − 0.39]
	*r = 0.48*, *p < 0.001*	*r* = − 0.54, *p* < 0.001
BSS total score		[− 0.58; − 0.27]
		*r* = − 0.44, *p* < 0.001

## Discussion

Main objectives of our study were to delineate time course of cough and wheezing in children with acute bronchitis and to validate the feasibility of automated, long-term cough- and wheezing monitoring in children. During the last years, some efforts have been made to establish automated or semi-automated cough monitoring systems, which can objectively record cough events [5, 6, 11, 26]. The clinical experience of using cough monitors to date is that they are practical and valid [29]. Primary application of all hitherto available cough monitors lies in the context of clinical trials [4]. Compared to other commercially available cough recording devices, LEOSound has the advantage that it can as well detect and analyze wheezing. Due to this long-term recording, time course of cough and wheezing can be evaluated objectively. The LEOSound monitor was studied in chronic obstructive pulmonary disease [9, 18], in cystic fibrosis and primary ciliary dyskinesia [24] in adults with asthma [17] and in patients with obstructive sleep apnea [31], respectively.

Reliable information about the expected course of respiratory tract infection in children is important for parents and clinicians. One of the most common questions that parents ask when consulting healthcare providers is “How long will the symptoms of acute bronchitis last?” In a systematic review by Thompson et al. (2013), expected durations of the most common respiratory symptoms in childhood respiratory tract infection are reported, including earache, sore throat, and cough. In randomized controlled trials, recovery of children with common cold ranged from 2 to 21 days. In observational studies, recovery lasted 2 to 3 weeks; mean duration of symptoms before trial entry ranged from 1 to 8.7 days. Outcome assessment was usually performed by parents using interviews or symptom diaries, or by the children themselves. Four clinical trials included 457 children and 15 observational studies all together included 4.870 children. Among the four clinical trials, common cold symptoms lasted 7 to 15 days. Based on pooled data from these four studies (Hutton et al. 1991, Macknin et al. 1998, Pappas et al. 2008, Smith et al. 2010), which reported proportions of children with symptoms by day 10, about 50% of children had improved by day 13 [14, 19, 23]. An additional study (Gruber et al. 2008) reported respiratory symptoms lasting 1.8 weeks (SD 1.3) in children aged 7 years or younger [13].

Automatic detection and classification of cough and wheezing is useful to assist physicians in diagnosing and monitoring respiratory diseases such as asthma and COPD [9, 18]. To the best of our knowledge, this is the first study in which cough and wheezing were recorded longitudinally in children with acute bronchitis. One primary study aim was to validate the feasibility of the LEOSound device to record and describe children’s bronchitis symptoms (cough and wheezing) in the course of acute bronchitis. Description of the general course of symptoms of bronchitis was a secondary aim. We could demonstrate continuous decrease of wheezing and cough within the first 9 days of bronchitis. From a clinical standpoint, this confirms findings of others, i.e., that cough can last 9 days (or more) in children with respiratory infection. Inclusion in our study was independent of prior therapy. Only nine out of 36 children did not have any medication. Thus, in terms of treatment, the participants in our study represent a heterogeneous group, and the measured time course of cough and wheezing may not apply uniformly, and may not be representative for untreated bronchitis. Future (better Future?) studies would require either an untreated or uniformly treated patient population for more generalizable study results. Nevertheless, this study could show that automated long-term lung sound monitoring is suitable and valid for description of symptoms in the course of acute bronchitis in children.

When listening to the lungs of a child with acute bronchitis, one can hear coughing and wheezing, depending on the severity of the mucous congestion and narrowing of the airways. Wheezes are high-pitched continuous adventitious sounds caused by airway narrowing which then cause airflow limitation. As shown in Fig. 3, cough was predominantly found at the beginning of the night. The time course of cough and wheezing frequency displayed opposite trends during each recording night. A possible explanation for this pattern could be that reduced coughing leads to an accumulation of secretion in the bronchi which consecutively contributes to airway narrowing and hereafter enhances wheezing. Within the three recording nights, cough frequency reduced to 35.3% of the magnitude measured at the beginning of the study. In the first night, 1299 cough epochs were recorded, in the second night (day 5) 490 and in the third night (day 9) 459.

We could show that productive cough decreases during the course of the study, while non-productive cough slightly increased during the last night of recording. Discrimination of cough in productive and non-productive events or epochs can help to validate if and how a certain medication acts. Hence, determination of type of cough could be a possible outcome parameter in clinical trials to prove effectiveness of treatments, e.g., secretolytic medication. The correlation between BSS-Score and PAC-QoL/Cough log-score showed that assessments of health status by parents and doctors are largely consistent. Therefore, severity of cough has a direct impact on quality of life. It has to be mentioned that in contrast to cough, nocturnal wheezing has not been considered in the validated questionnaires. Since wheezing sounds are quiet and can hardly been heard without auscultation devices, these sounds cannot be documented or scored by parents. Hence, assessment and evaluation of wheezing events require objective approaches. Lung sound analysis has shown to be the most appropriate method to objectively record these events [3, 8].

Long-term recording of lung sounds is a helpful instrument to analyze time course of respiratory symptoms in patients with bronchitis. End points for future clinical trials could be cough frequency, number and duration of cough epochs, characterization of cough into productive and non-productive and duration of wheezing. In the future, lung sound analysis could be an auxiliary method for gaining valid and comparable data regarding frequency of respiratory symptoms in acute bronchitis, especially for evaluation of clinical effectiveness of commonly used protussive and antitussive cough remedies.

## Abbreviations

PAC-QoL: Parent-proxy Children’s Acute Cough-specific Quality of Life
BSS: Bronchitis Severity Scale
